# The Relationship Between the Index of Cardio‐Electrophysiological Balance and the Non‐Dipper Hypertensive Pattern in Patients With Newly Diagnosed Hypertension

**DOI:** 10.1111/jch.70196

**Published:** 2026-01-14

**Authors:** Seda Tükenmez Karakurt, Hüseyin Karakurt, Nail Güven Serbest, Serkan Yazan

**Affiliations:** ^1^ Department of Cardiology M.D. Mehmet Akif Ersoy Thoracic and Cardiovascular Surgery Training Research Hospital Istanbul Turkey

**Keywords:** ambulatory blood pressure monitoring, circadian blood pressure, hypertension, index of cardio‐electrophysiological balance, non‐dipper hypertension

## Abstract

The connection between different electrocardiography (ECG) parameters and circadian blood pressure (BP) variations in patients with hypertension (HT) has been investigated. The index of cardio‐electrophysiological balance (iCEB), determined as the quotient of the QT interval and the QRS duration, offers an assessment of the comprehensive equilibrium between depolarization and repolarization. This study aimed to explore the relationship between iCEB and circadian BP variability.

After applying exclusion criteria, a total of 144 individuals were diagnosed with HT based on the 24 h ambulatory blood pressure monitoring (ABPM) results. Using the results from 24 h ABPM, the study participants were divided into two groups: those with dipper HT and those with non‐dipper HT. The iCEB is calculated by dividing the QT interval by the QRS duration (QT/QRS).

The iCEB was significantly higher in individuals with non‐dipper HT compared to those with dipper HT. (3.88 ± 0.6 vs. 4.38 ± 0.89 respectively, *p* < 0.001). Univariate logistic regression analysis revealed significant correlations between non‐dipper hypertensive pattern and creatinine, frontal QRS‐T angle (FQRSTA), and iCEB. As a result of multivariate analysis, iCEB (OR:3.125, 95% CI: 1.595–6.117; *p* = 0.001) was found to be an independent predictor of non‐dipper HT. iCEB optimal cut‐off value of > 4.1 predicted non‐dipper hypertensive pattern with 67.4% sensitivity and 67.3% specificity.

This study indicated that a higher iCEB was linked to non‐dipper HT in newly diagnosed hypertensive patients.

## 1 Introduction

Ambulatory blood pressure (ABP) monitoring (ABPM) has gained growing significance in managing patients with hypertension (HT) [1]. Most research indicates that the average 24 h ABPM provides a more accurate prediction of morbidity and mortality compared to office blood pressure (BP) [2]. Although hypertensive patients are characterized by elevated intravascular pressure, disturbances in circadian BP patterns have been demonstrated to potentially result in unfavorable clinical outcomes [3]. This is because disrupted circadian BP variations are important predictors of end‐organ damage and a higher likelihood of adverse cardiovascular events in the long term [4]. BP usually exhibits a consistent circadian rhythm, dropping by 10–20% at night compared to daytime, governed by natural neuroendocrine fluctuations and other influences. Dipper HT is defined by a decrease of over 10% in both systolic and diastolic BP during nighttime. Conversely, non‐dipper HT is characterized by a less than 10% reduction in these blood pressure readings [5].

Traditional markers of hypertensive cardiac involvement—such as electrocardiographic left ventricular hypertrophy (LVH) criteria, echocardiographic left ventricular mass index (LVMI), and relative wall thickness (RWT)—provide structural information reflecting chronic hypertensive remodeling. However, newly diagnosed, treatment‐naïve patients often lack overt structural changes.

The connection between different electrocardiography (ECG) parameters and circadian BP variations in patients with HT has been studied [6, 7, 8]. The index of cardio‐electrophysiological balance (iCEB), determined as the quotient of the QT interval and the QRS duration, offers an assessment of the comprehensive equilibrium between depolarization and repolarization. An elevated iCEB signifies a disproportionate extension of repolarization in comparison to depolarization, which correlates with an increased risk of arrhythmias [9]. In the scholarly literature, although iCEB has been documented to be linked with the prediction of arrhythmia risk in hypothyroid patients [10], drug‐induced cardiac arrhythmias [9], and the forecasting of major adverse cardiac events as well as cardiovascular mortality in diabetic patients with associated cardiovascular disease [11], its correlation with HT patients, particularly concerning circadian BP variations, has not been extensively investigated or reported. Although the QT interval and QRS duration, components of iCEB in hypertensive patients, have been individually examined in relation to prognosis and circadian BP fluctuations, their combined association with circadian BP variations has not yet been documented in the literature. Therefore, this study aimed to explore the connection between iCEB and circadian BP variability.

## 2 Methods

### 2.1 Study Participants

**FIGURE 1:**
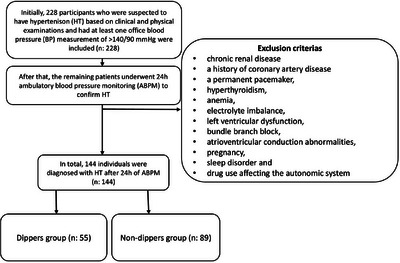
Flowchart.

Initially, 228 participants suspected of having HT based on clinical and physical examinations, each of whom had at least one office BP measurement exceeding 140/90 mmHg, were included. Patients with chronic renal disease, a history of coronary artery disease, a permanent pacemaker, hyperthyroidism, anemia, electrolyte imbalance, left ventricular dysfunction, bundle branch block, atrioventricular conduction abnormalities, pregnancy, sleep disorders, or drug use affecting the autonomic system were excluded from the study. The remaining patients then underwent 24 h ABPM to confirm HT. In total, 144 individuals were diagnosed with HT based on the 24 h ABPM results (Figure 1: flowchart). For each patient enrolled in the study, their medical history and laboratory measurements were recorded. Office HT was defined as a systolic BP exceeding 140 mmHg and/or a diastolic BP exceeding 90 mmHg. The study was conducted in full accordance with the Helsinki Declaration, following approval from the local ethics committee.

## 3 24 h Ambulatory Blood Pressure Recordings

The current guidelines were used to diagnose HT with an ABPM device. (SunTech Medical Inc., Morrisville, USA) [1]. 24 hABPM recordings were taken every 15 min during the day and every 30 min at night, with awake and sleep periods determined from patient data. Nocturnal BP dipping was calculated using the formula: (%) 100 × [1– (sleep systolic BP / awake systolic BP)]. Based on this dipping, circadian BP patterns are classified as dipper (0.8 < ratio ≤ 0.9) and non‐dipper (0.9 < ratio ≤ 1.0) [5]. HT on ABPM was diagnosed using ESC‐2024 thresholds: 24 h ≥ 130/80 mmHg, daytime ≥ 135/85, nighttime ≥ 120/70 [12].

### 3.1 Electrocardiographic and Echocardiographic Evaluation

**FIGURE 2:**
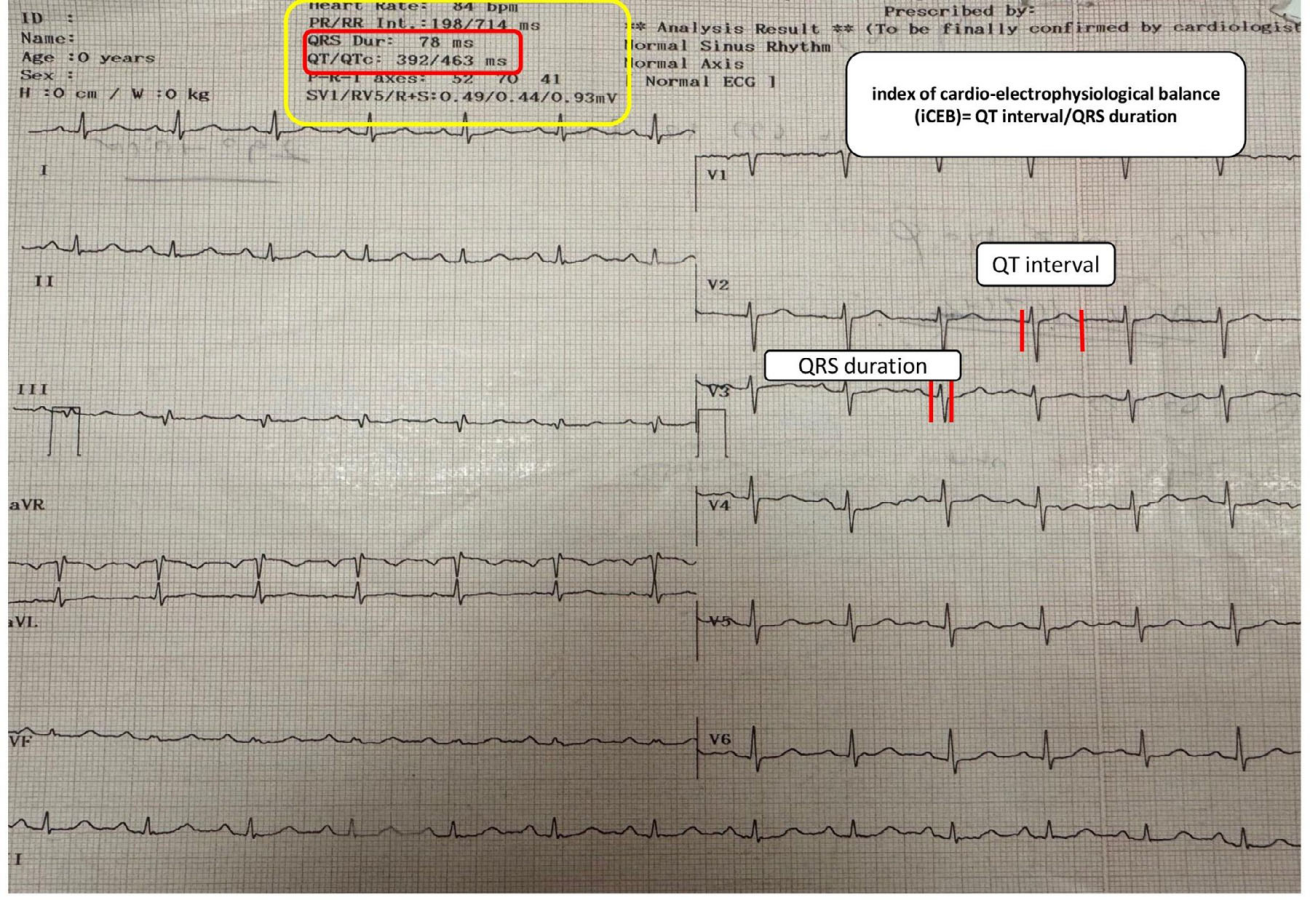
An example of the evaluation of the index of cardio‐electrophysiological balance from the 12‐lead electrocardiography with its automatic report.

All patients had a 12‐lead ECG (Nihon Kohden, Tokyo, Japan) recorded at rest while supine, using a speed of 25 mm/s, a voltage of 10 mm/mV, and a filter range of 0.16–100 Hz. The iCEB is calculated by dividing the QT interval by the QRS duration (QT/QRS), measuring the time between depolarization and repolarization processes (Figure 2). The frontal QRS‐T angle (FQRSTA) was simply determined by subtracting the QRS angle from the T angle on the ECG. If this difference was greater than 180°, it was adjusted by subtracting the value from 360° [13]. The Tp‐e interval was recorded in precordial leads. For complex T waves (such as biphasic or triphasic), the interval was measured from the nadir of the first component to the end of the T wave [14]. The duration of the QT interval was measured as the interval from the onset of the QRS complex to the point at which the T‐wave merges with the isoelectric line (Figure 2). The corrected QT interval was calculated utilizing Bazett's formula [15]. QT dispersion (QTd) was calculated by subtracting the minimum QT duration from the maximum QT duration [15]. QT and QRS durations were automatically calculated by the ECG device. QT and QRS intervals were obtained from automated ECG software and visually verified. The iCEB ratio, derived from the QT/QRS, was calculated by an independent cardiologist, blinded to the 24 h ABPM results, analyzed all ECG parameters. Figure 2 provides an example of the automated iCEB evaluation from the 12‐lead ECG.

Transthoracic echocardiography was conducted on all patients using a Philips HD 11 XE ultrasound system (Andover, MA, USA). All measurements adhered to the American Society of Echocardiography guidelines [16]. An experienced cardiologist performed all echocardiographic evaluations. In a random sample of 30 patients, the intraclass correlation coefficients for ejection fraction (EF), interventricular septum (IVS), left ventricular end diastolic diameter (LVEDD), and left ventricular end systolic diameter (LVESD) measurements were each greater than 95%. Although traditional markers such as electrocardiographic LVH criteria and echocardiographic indices of hypertensive organ damage (e.g., LVMI, RWT) are well‐established prognostic tools, our study population consisted of newly diagnosed and treatment‐naïve hypertensive individuals without structural heart disease or LVH. Therefore, these parameters were not expected to differ significantly between groups.

### 3.2 Statistical Analyses

Statistical analyses were conducted using SPSS version 22.0 (SPSS Inc., Chicago, IL, USA). Continuous variables were presented as mean ± standard deviation or median (interquartile range [IQR] 25–75), depending on their normality and distribution. These were compared with variance analysis (for parametric data) or Kruskal–Wallis *H* test (for non‐parametric data). Categorical variables were shown as counts and percentages (%) and compared using the *χ*2 test or Fisher's exact test. Variables significantly associated with BP pattern in univariate analysis were incorporated into a multinomial logistic regression model with a backward elimination approach (*p* < 0.1). ROC curve analysis assessed the iCEB's ability to predict non‐dipper status. Significance was indicated by the odds ratio (OR) with 95% confidence intervals (CI). A *p* value < 0.05 was deemed statistically significant.

## 4 Results

The study included 144 newly diagnosed hypertensive patients with an average age of 53 ± 11 years; 84 (58.3%) of patients were female. Among these, 55 exhibited a dipper BP pattern, while 89 showed a non‐dipper BP pattern.

Table 1 delineates the baseline clinical characteristics, 24 h ABPM data, laboratory findings, and ECG and echocardiographic features of the study cohort. No statistical differences were identified between the groups concerning age, gender, body mass index, smoking status, or the presence of diabetes mellitus. Although not reaching statistical significance, the female gender was most frequently observed in both the dipper and non‐dipper groups. The non‐dipper group exhibited the highest nocturnal systolic and diastolic blood pressures. Creatinine levels in non‐dipper hypertensive patients were significantly higher compared to those in dipper patients, although this did not seem to be clinically significant. No significant differences were observed between the groups concerning echocardiographic parameters.

**TABLE 1:** The baseline clinical characteristics, 24 h ambulatory blood pressure monitoring, laboratory results, echocardiographic, and electrocardiographic features of the study population.

	Dippers (*n*: 55)		Non‐dippers (*n*: 89)		All patients (*n*: 144)		*p*
Age (years)	53	± 11	53	± 10	53	± 11	0.799
Sex, *n* (%) (Female)	29	(52.7)	55	(61.8)	84	(58.3)	0.285
BMI (kg/m^2^)	26.62	± 1.26	26.77	± 1.23	26.71	± 1.24	0.345
Smoking, *n* (%)	11	(20)	24	(27)	35	(24.3)	0.714
Diabetes, *n* (%)	8	(14.5)	15	(16.9)	23	(16)	0.309
Hemoglobin (g/DL)	14.5	± 1.7	14.8	± 1.6	14.7	± 1.6	0.225
Creatinine (mg/dL)	0.71	± 0.18	0.78	± 0.19	0.75	± 0.19	**0.011**
Glucose (mg/dL)	101	± 20.4	104.5	± 31.3	103.2	± 27.6	0.765
Systolic BP (total) (mmHg)	142	± 16	145	± 14	144	± 15	0.156
Diastolic BP (total) (mmHg)	86	± 13	87	± 11	87	± 12	0.394
Systolic BP (awake) (mmHg)	145	± 24	145	± 21	145	± 22	0.651
Diastolic BP (awake) (mmHg)	90	± 13	88	± 12	89	± 12	0.63
Systolic BP (sleep) (mmHg)	130	± 15	144	± 16	138	± 17	**0.001**
Diastolic BP (sleep) (mmHg)	77	± 12	85	± 12	82	± 13	**0.001**
EF (%)	64	± 5	65	± 3	65	± 4	0.129
IVS (mm)	10.7	± 1.3	11.2	± 1.7	11	± 1.5	0.251
LVEDD (mm)	49	± 3.7	48	± 4.4	48	± 4.1	0.21
LVESD (mm)	33	± 5	32	± 5	33	± 5	0.258
Heart Rate (bpm)	78	± 13	79	± 13	79	± 13	0.422
Tp‐e interval (msn)	67	± 14	70	± 12	69	± 13	0.193
Tp‐e/QT interval	0.17	± 0.04	0.18	± 0.03	0.18	± 0.04	0.389
QTd (msn)	53	(40–70)	57	(40–66)	56	(40–69)	0.956
FQRST Angle (°)	22	(14–44)	43	(21–66)	34	(18–57)	**0.001**
QT inteval (msn)	384	± 26	396	± 33	391	± 31	**0.038**
QTc (msn)	435	(432–454)	430	(405–454)	433	(409–456)	0518
QRS Duration (msn)	97.43	± 17.69	94.81	± 14.09	95.74	± 15.45	**0.04**
iCEB	3.88	± 0.6	4.38	± 0.89	4.19	± 0.83	**<0.001**

Abbreviations: BP, blood pressure; BMI, body mass index; EF, ejection fraction; IVS, interventricular septum; LVEDD, left ventricular end diastolic diameter; LVESD, left ventricular end sistolic diameter; Tp‐e, T peak T end; FQRST, frontal QRS‐T; QTd, QT dispersion; QTc, corrected QT; iCEB, index of cardio‐electrophysiological balance.

The iCEB was significantly higher in individuals with non‐dipper HT compared to those with dipper HT. (3.88 ± 0.6 vs. 4.38 ± 0.89 respectively, *p* < 0.001). Furthermore, the QT interval, a sub‐parameter of iCEB, was observed to be elevated in the non‐dipper group, whereas the QRS duration was increased in the dipper group.

Univariate logistic regression analysis revealed significant correlations between non‐dipper hypertensive pattern and creatinine, FQRSTA, and iCEB. As a result of multivariate analysis, iCEB (OR: 3.125, 95% CI: 1.595–6.117; *p* = 0.001) was found to be an independent predictor of non‐dipper HT (Table 2).

**TABLE 2:** Independent predictors of non‐dipper hypertension multivariate logistic regression analysis.

	Univariate			Multivariate		
	Univariate OR, 95% CI		*p*	Multivariate OR, 95% CI		*p*
**Creatinine**	11.459	(1.405–93.485)	**0.023**	20.87	(1.155–28.1)	**0.04**
**FQRSTA**	1.026	(1.0–1.045)	**0.001**	1.028	(1.011–1.045)	**0.002**
**iCEB**	2.883	(1.612–5.156)	<**0.001**	3.125	(1.595–6.117)	**0.001**

Abbreviations: OR, odds ratio; CI, confidence interval; FQRSTA, frontal QRS‐T angle; iCEB, index of cardio‐electrophysiological balance.

**FIGURE 3:**
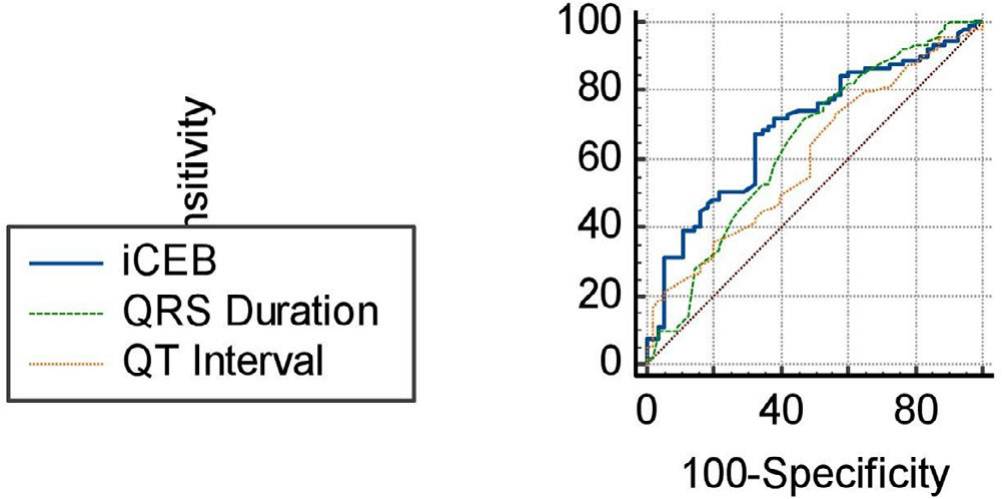
Receiver operating characteristic (ROC) curve analysis of the index of cardio‐electrophysiological balance for predicting non‐dipper hypertensive pattern.

iCEB optimal cut‐off value of > 4.1 predicted non‐dipper hypertensive pattern with 67.4% sensitivity and 67.3% specificity ([AUC]: 0.691 [95% CI: 0.658–0.764, *p* = 0.04]) (Figure 3).

To determine if iCEB provided extra predictive value beyond components such as QT and QRS, their ROC curves were compared. The AUC value of iCEB (AUC: 0.691 [95% CI: 0.658–0.774, *p* = 0.04]) was found to be significantly higher than that of QT (AUC: 0.603 [95% CI: 0.518–0.683, *p* = 0.047]) and QRS (AUC: 0.643 [95% CI: 0.559–0.721, *p* = 0.048]) in predicting non‐dipper hypertensive pattern (Figure 3).

## 5 Discussion

The main finding of this study is that the iCEB was significantly higher in patients with non‐dipper HT compared to those with dipper HT. Additionally, an elevated iCEB independently predicted non‐dipper HT in newly diagnosed hypertensive patients. Patients with non‐dipper BP profiles demonstrated higher iCEB values, suggesting early electrophysiological imbalance. The high prevalence of non‐dippers reflects the characteristics of untreated, newly diagnosed hypertensive patients. Some non‐dippers exhibited borderline reverse‐dipping patterns, possibly due to nocturnal sympathetic activation or poor sleep quality.

Daytime and nighttime ABP are significant predictors of all‐cause and cardiovascular mortality, as well as cardiovascular disease (CVD), stroke, and a combined measure of major CVD, independently of office BP and other confounding variables. Additionally, only nighttime ABP predicts non‐cardiovascular mortality [3]. Daytime ABP significantly and independently predicted all‐cause mortality, cardiovascular (CV) mortality, and stroke mortality in patients referred for ABPM [17, 18]. It also predicted all strokes in older hypertensive patients and an increase in cardiovascular events in cases of refractory hypertension. Nighttime ABP independently forecasted all‐cause, CV, and stroke mortality, but not total stroke [19]. When both daytime and nighttime ABP were analyzed together for mortality prediction, nighttime ABP proved to be more effective than daytime ABP in predicting all‐cause, CV, cardiac, and stroke mortality [17]. Therefore, it is crucial to identify non‐dipper hypertensive individuals early in the course of HT [20]. The decrease in circadian BP variability mainly stems from reduced activity of the autonomic nervous system. Previous research shows that people with disrupted circadian BP regulation have an imbalance between sympathetic and vagal activity [21].

The connection between different ECG parameters and circadian BP fluctuations in patients with HT has been studied [6, 7]. ICEB can be represented by the QT/QRS ratio, which is analogous to the traditional *λ* value (*λ* = effective refractory period × conduction velocity). The QRS duration reflects changes in conduction velocity, while variations in QT intervals relate to shifts in the effective refractory period (ERP). Therefore, using ICEB as a surrogate for *λ* not only circumvents the need for invasive measurements but also helps predict the risk of TdP and non‐TdP ventricular tachycardia or fibrillation [11]. Although iCEB has been documented to be linked with the prediction of arrhythmia risk in hypothyroid patients [10], drug‐induced cardiac arrhythmias [9], and major adverse cardiac events as well as cardiovascular mortality in diabetic patients with associated cardiovascular disease [11], its correlation with HT patients—particularly concerning circadian BP variations—has not been extensively investigated or reported. To the best of our knowledge, this is the first study to assess the iCEB in patients with HT, making our findings a meaningful contribution to understanding the circadian BP changes associated with this marker. An increased iCEB level reflects a disproportionate prolongation of repolarization compared to depolarization, providing a comprehensive view of these cardiac cycle phases [9]. Previous research showing higher frontal QRS‐T angles (FQRSTA) linked to greater ventricular repolarization heterogeneity supports our conclusion that HT has a significant impact on cardiac electrophysiology [7, 20]. In our study, FQRSTA was found to be higher in non‐dipper HT patients, consistent with existing research.

In our study, creatinine levels were notably higher in non‐dipper hypertensive patients compared to dipper patients, although this difference did not appear to be clinically meaningful. Although certain studies have not demonstrated a significant correlation between renal function and circadian BP variations, Sun L et al. reported renal impairment and elevated creatinine levels, particularly in non‐dipper hypertensive patients compared with dipper hypertensive patients [22]. We believe that further research is warranted to explore the relationship between impaired circadian blood pressure regulation, creatinine levels, and renal function.

Research indicates that myocyte hypertrophy and collagen deposition in the interstitium start during the prehypertensive stage. Consequently, the uniform structure of the left ventricle becomes disrupted, and fibrotic tissue accumulates [23]. This results in an alteration of myocardial repolarization. [24]. Cardiac magnetic resonance imaging shows that patients with non‐dipper HT are more prone to exhibit late gadolinium enhancement than those with dipper HT. This indicates that higher nocturnal BP is linked to increased fibrosis in the myocardium [25, 26]. This also explains why iCEB, which reflects changes in repolarization and neuro‐autonomic imbalance, was more prominent in non‐dipper HT in our study. The research demonstrated that iCEB independently predicts non‐dipper HT.

### 5.1 Limitations

Our study has several limitations. First, the sample size was small, and we included only individuals recently diagnosed with HT. Second, manual measurement of ECG parameters can lead to observer bias, but standardized protocols were implemented to minimize this bias. Third, although iCEB demonstrated independent predictive value, its discriminative accuracy is modest; therefore, it should be interpreted as an adjunctive—not standalone—marker. Consequently, it may be difficult to generalize these results to those who have been diagnosed and treated for longer periods.

## 6 Conclusion

Our study found that a higher cardio‐electrophysiological balance index (iCEB) is associated with non‐dipper hypertension (HT). Since iCEB is a simple and easy‐to‐measure ECG parameter, it could serve as an additional diagnostic tool to identify non‐dipper blood pressure (BP) patterns in patients who are newly diagnosed with HT.

## Author Contributions

S. T. K., H. K., N. G. S., and S. Y. have significantly contributed to the conception, design, data collection, statistical analysis, and drafting of the manuscript. All authors participated in data analysis and manuscript drafting, and have read and approved the final version.

## Funding

The authors received no specific funding for this work.

## Conflicts of Interest

The authors declare no conflicts of interest.

## Data Availability

The authors have nothing to report
